# Discussions of Flavored ENDS Sales Restrictions: Themes Related to Circumventing Policies on Reddit

**DOI:** 10.3390/ijerph19137668

**Published:** 2022-06-23

**Authors:** Nathan Silver, Padmini Kucherlapaty, Ganna Kostygina, Hy Tran, Miao Feng, Sherry Emery, Barbara Schillo

**Affiliations:** 1 Truth Initiative Schroeder Institute, Washington, DC 20001, USA; pkucherlapaty@truthinitiative.org (P.K.); bschillo@truthinitiative.org (B.S.); 2 NORC at the University of Chicago, Chicago, IL 60603, USA; kostygina-anna@norc.org (G.K.); tran-hy@norc.org (H.T.); feng-miao@norc.org (M.F.); emery-sherry@norc.org (S.E.)

**Keywords:** advertising and promotion, tobacco industry, media

## Abstract

Objective: To examine conversations among JUUL users on Reddit related to restrictions on flavored ENDS and the shifting policy landscape. Methods: Posts and comments (*n* = 166,169) between May 2019 and May 2020 on the subreddit r/JUUL were scraped using pushshift.io API. Keyword filters were used to identify texts discussing flavored ENDS products (*n* = 33,884 texts). These were further narrowed down to texts discussing flavor policy workaround strategies (*n* = 7429) and N-gram analysis was performed. Finally, findings from the N-gram analysis were triangulated through qualitative review of a separate sample of texts (*n* = 488) from the flavor policy-related posts and comments. Results: Overall activity on the subreddit r/JUUL peaked around the time of the EVALI outbreak (September 2019) and when FDA issued guidance restricting flavored ENDS product sales (January 2020). The N-gram analysis revealed an active discussion of banned products one can “still get” or “JUUL compatible” alternatives, including specific brands, brick and mortar locations, and specific flavors. Ten dominant themes emerged from the qualitative review, with some posts containing more than one theme. Conclusion: Many users turned to Reddit for information related to the shifting regulatory landscape concerning flavored ENDS. Discussions focused on both legal alternatives to banned products as well as illegal means of acquiring JUUL pods, including residual retail supply, online, and mail vendors.

## 1. Introduction

Ongoing efforts to restrict the availability of flavored tobacco products necessitates careful examination of the public response to a rapidly evolving electronic nicotine delivery systems (ENDS) marketplace. In the wake of the e-cigarette or vaping use-related lung injury (EVALI) outbreak in September 2019, electronic cigarette manufacturer JUUL voluntarily removed flavored pods aside from tobacco and menthol from the U.S. market [1]. JUUL’s voluntary withdrawal was followed up by FDA guidance in January 2020 which removed all flavored pod-based ENDS from the marketplace [2]. At the time, JUUL was the most popular reusable e-cigarette on the market, attracting a large number of users through their flavored and high nicotine strength products [3,4]. This shift in the policy landscape resulted in bare shelves inevitably leaving users of JUUL’s popular flavored pods with questions and motivation to find alternative products.

Flavored Tobacco Product (FTP) policies play a critical role in reducing the access and availability of these products [5], particularly given the tobacco industry’s historical targeting of youth using flavored products that are easier to initiate and thus more likely to lead to sustained use [6]. However, unintended consumer adaptations to FTP restrictions such as switching to alternative products or identifying alternative supply sources can undermine the effectiveness of such policies [7]. As a result, continuous evaluation of consumer responses to policy changes is an essential component of evidence-based policy making [8]. Thus, examining JUUL pod user responses during the course of policy implementation can inform how to optimize the policy and its public health benefits [9].

Since the initial ban on flavored pods, many users simply switched to alternative flavored products [10]. Bans that were initially imposed locally led to increased internet sales and cross-border purchases of the banned flavors [11]. JUUL users reported using generic pods or disposable products, stockpiling banned flavors, and purchasing banned flavors from stores that illegally sold them [11,12]. Moreover, new disposable flavored products took over JUUL’s market share, exposing a loophole wherein such disposable devices were not subject to the flavor restrictions as they were technically different products [13]. Such loopholes in the sales environment and the potential for substituting products undermined the intention of the new FTP policy in addition to leaving unanswered questions for users navigating a sudden absence of their preferred product.

The abrupt change in the product landscape caused by the flavored pod ban likely lead users to rely on social media platforms for up-to-date information. Changes at the societal level, such as a shift in policy banning a popular product, can lead people to mediated channels [14], particularly social media [15,16], to understand the implications of such changes on their lives. Given inconsistent information about ENDS in general has been shown to drive users to social media [17], we suggest that such a dramatic shift in the ENDS product landscape would lead JUUL users to social media to investigate and understand how the flavored pod-based ENDS ban would affect them. Because policy and regulation is one of the major themes discussed on ENDS-related social media, analyzing such discourse around the time of policy implementation can offer useful and unobtrusive access to public sentiment and response to regulatory action [17]. We can leverage these immediate responses and resulting changes in use behaviors to identify gaps and address them in future policy iterations.

Not surprisingly, a robust community of JUUL users on Reddit, a popular moderated, forum-based social media platform, provided lots of discussion about the new FTP restrictions, as users sought information regarding how the newly imposed ban affects them, as well as instrumental support regarding the residual availability of newly banned products, replacement alternatives, and other means of adjusting to or even evading the consequences of the new flavor policy. Examining these discussions can help to evaluate the effectiveness and limitations of this policy. Though by no means immune to the limitations of data from other social media platforms, posts from Reddit are often detailed and candid and focus on shared experiences rather than punchy sound bites [18], and thus may be better suited to capture the nuances of day-to-day experiences related to navigating a changing product landscape. Reddit has been used in recent years to track the popularity of various flavors, underage use, and even potential effects of flavored ENDS products [19,20,21,22,23].

This study aims to leverage Reddit discussions among JUUL users to better examine how they responded to real-time changes in FTP policies. Although the rise in popularity of disposable flavored products is likely the direct result of the most recent wave of FTP policies [10], understanding how users of the affected products leverage informal communication channels such as Reddit during such a transitionary period can potentially help to more effectively anticipate unintended consequences of future FTP policies.

## 2. Materials and Methods

### 2.1. Procedure

To examine how users of flavored ENDS responded to shifting FTP policies, we collected, parsed, and analyzed posts and comments from r/JUUL using the third-party archive of Reddit content, pushshift.io API, to scrape all posts and comments between 1 May 2019 and 31 May 2020. Although we were primarily interested in the time period from August 2019 to January 2020, which included EVALI, JUUL’s voluntary removal of flavored pods, and the FDA’s guidance to ban flavored pods, we gathered the whole year of data to place these events within the context of the whole year. Two keyword filters were validated and used to narrow the total sample of texts down to those related to flavors and then further to those associated with FTP policies. Analyses of post and comment volume over time, N-gram analysis of relevant content identified by our keyword filters, and qualitative analysis of content identified by human coders were used to systematically examine the most common responses to the recently imposed regulations. All validated search parameters are available in the Supplementary Table S1.

### 2.2. Data Acquisition and Classification

An initial corpus of *n* = 166,169 texts (posts and comments) was retrieved from r/JUUL for further classification. Keyword filters were first used to identify texts discussing flavored ENDS products, and then further filtered to identify FTP policy-related content. For the flavor filter, *n* = 2500 posts were dual coded for correct identification of flavored ENDS product relevance (kalpha = 0.92). The filter performed well in accurately identifying flavor-related content (precision = 0.87, recall = 1, F1 = 0.93). This initial filter yielded a flavor-related corpus of *n* = 33,884 texts. We then used a keyword filter to identify policy-related texts from within the flavor-related corpus. To evaluate the accuracy of this keyword filter, *n* = 200 posts were dual coded for the correct identification of relevant content. The coders were reliable (kalpha = 0.88), and the filter was sufficiently accurate (precision = 0.88, recall = 0.76, F1 = 0.82). N-gram analysis was then performed on *n* = 7429 texts that our filters categorized as about FTP policies to provide insight into the content of these discussions through identifying the most common one, two, and three-word phrases.

To triangulate findings from our N-gram analysis, we randomly sampled *n* = 2000 texts from the 33,884 flavor-related texts for human coding. Two independent coders reliably (kalpha = 0.88) identified *n* = 488 texts related to FTP policy workarounds which were set aside for qualitative examination. All discrepancies were adjudicated by the study’s lead analyst. This extra round of human coding was performed to assure that qualitative examination would be conducted only on relevant content. In doing so, we both corroborate and add context and nuance to findings from our machine-driven approach.

### 2.3. Analyses

Three sets of analyses were conducted to further examine our data. First, using our filters, we graphed text volume by month for flavor and FTP policy-related content as a proportion of total content on the r/JUUL subreddit to visualize the relationship between the inciting event, EVALI, the ensuing ban on flavored pod devices, and the resulting discussion on Reddit. Next, we identified the most frequent words, bigrams, and trigrams (two and three word sets) among the *n* = 7429 texts identified by our relevance filters to better understand the content of FTP policy-related discussions. Finally, to triangulate inferences based on N-gram analysis, we qualitatively examined a subsample of *n* = 488 texts identified by human coders as about FTP work around strategies.

## 3. Results

### 3.1. Temporal Analysis through Visualization

**Figure 1:**
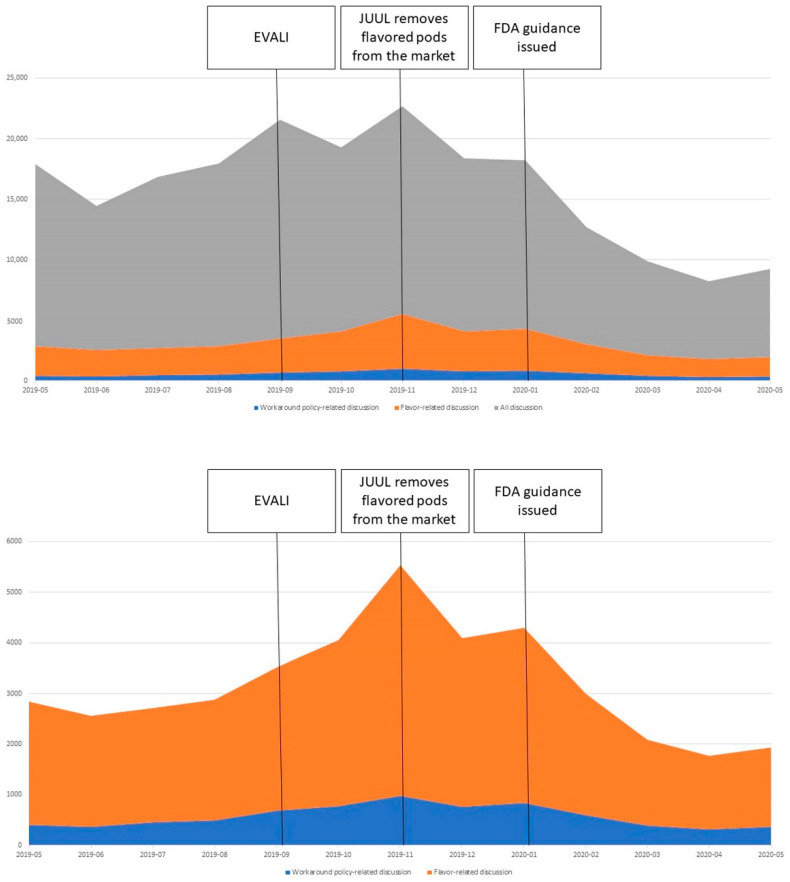
Temporal graphs comparing trends of the volume of activity on r/JUUL related to flavors and strategies to evade flavored product restrictions as well as all activity on the subreddit. (**Top**) FTP policy workaround discussion (blue) as a portion of flavor discussion (orange) as a portion of all activity on the subreddit (gray). (**Bottom**) The all activity (gray) portion is removed to better visualize the variation in FTP policy workaround discussion (blue) as a portion of all flavor-related discussion (orange).

Figure 1 provides a visualization contextualizing FTP policy workaround discussion during the five-month period, including the EVALI outbreak in September 2019, the subsequent halting of flavored pod sales by JUUL, and culminating in FDA guidance to remove flavored pods from the market in January 2020, within overall activity on the r/JUUL subreddit from May 2019 to May 2020. Overall activity on the JUUL subreddit increased consistently beginning in June of 2019, with a sharp increase and initial peak coinciding with media coverage of EVALI [24,25], reaching a zenith in November 2019 shortly after JUUL announced voluntary removal of its flavored pods from the market, and then declining sharply beginning January 2019 following the FDA’s guidance to remove all flavored pods from the market. General flavor discussions followed a similar pattern, minus the initial peak associated with EVALI. Finally, although not as dramatic as the peaks in the two broader categories, FTP policy workaround discussion steadily increased from May through November of 2019, peaking in November. A second peak in late January coincided with the FDA’s announcement.

### 3.2. N-Gram Analysis

Table 1 provides the most common words, bigrams, and trigrams suggesting an active discussion of what sort of pods one can “still get”, whether there were “JUUL compatible” alternatives including specific brands (e.g., Mr. Fog Pods) and brick and mortar locations (e.g., gas station, local vape shop), presumably in an effort to locate specific pod flavors (e.g., mint, mango). Phrases such as “black market THC” and “black market pods” indicate significant discussion of illicit markets, though N-gram analysis does not make clear whether these conversations were in reference to the role of illicit THC (i.e., tetrahydrocannabinol) cartridges in the EVALI outbreak, active attempts to gain access to illicit sources of newly unavailable pods, or a combination of the two.

### 3.3. Qualitative Analysis

The qualitative review revealed ten dominant themes across workaround-related content, with some posts containing more than one theme. More than one in three posts (39%) were seeking advice on disposable or refillable substitute brands that offered newly unavailable flavors, including 5.9% which named specific refillable pod device brands including Caliburn (9), Smok (7), Voopoo (1), Novo (7), and Vuse (5). One post mentioned OVNS, a refillable pod brand that is compatible with JUUL devices. Advice on product availability in localities where the ban had recently gone into effect (e.g., Massachusetts, Michigan, etc.) was sought in 23% of posts. Furthermore, 20% of posts inquired about how to acquire products online, while 16% concerned the ability to import products from abroad (e.g., Canada, Russia, and UK). Other topics were present in fewer than 5% of reviewed posts. For example, 4% discussed stockpiling or bulk buying and 2.5% sought advice on counterfeit, third-party, and off-brand pods. Fewer than 1% of posts concerned petitioning authorities regarding the newly imposed regulations, substituting other tobacco products including cigarettes and smokeless products, obtaining products from friends or family, and modifying unflavored pods using accessories or “slip-tips”. Thematic analysis reaffirmed findings from the N-gram analysis showing an overwhelming majority of discussions related to the newly imposed flavor restrictions were focused on the availability of alternatives and how to obtain them. Discussions of a “black market” identified in N-gram analysis were revealed to focus on the role of illicit market THC pods in the EVALI outbreak, decrying that a ban on flavored pods would inevitably lead to an illicit market, as well as seeking potential illicit market sources. The prominence of “mango” and “mint” in our N-gram analysis was indicative of the two most popular JUUL pod flavors [26] also being the most sought-out in FTP policy workaround-related discussions on Reddit.

## 4. Discussion

This study describes the content of discussions by flavored JUUL pod users to the evolving flavored product restrictions in real time. Significant spikes in usage of the r/JUUL subreddit corresponding to EVALI, JUUL’s voluntary removal of flavored pods, and again following issuance of FDA guidance suggest that the r/JUUL subreddit is a popular online platform for users to discuss product and policy shifts related to ENDS. The period between the EVALI outbreak and the FDA guidance removing flavored pods from the market saw a substantial increase in both overall activity on the subreddit as well as discussions related to the implications of the flavor ban. Qualitative review of these posts revealed that 95% of them concerned either alternative means of acquiring newly banned flavors or soliciting similar alternative products. Although there was some discussion of illicit markets, most discussions blamed the illicit market of THC cartridges for the new restrictions on JUUL pods. These findings highlight both the utility of Reddit for evaluating the public’s response and the potential limitations of newly enacted FTP policy.

The findings also highlight the need for comprehensive flavor restrictions given the almost instantaneous response of JUUL pod users to identify alternative flavored products. Although JUUL was credited with the popularity of ENDS with youth, disposable flavored ENDS brands quickly filled the void left by JUUL pods [10]. Discussions about alternatives and residual supply were among the most common topics of discussion related to the new flavor restrictions. It is noteworthy that two of the brands identified in our qualitative analysis as alternatives include Vuse and Smok, which were identified as two of the four most popular brands with youth along with JUUL and puff bar [27]. Consistent with previous research [28,29], the implications of this quick response to identify alternative products strongly indicates flavors as a crucial component for using ENDS products. Thus, effective flavor policy should focus not only on the specific products currently used by youth and young adults [30], but rather focus more broadly on anticipating the consumer response and market innovations by restricting all flavored products including low-cost-of-entry flavored pod devices and disposables.

Although discourse surrounding tobacco regulation spans a variety of social media platforms, Reddit may be of particular use in examining more in-depth discussions. Reddit currently has over 440 million active users [31], but most importantly allows for long posts on moderated, topic-specific forums called subreddits, which can be discussed in depth by an anonymous user-base. Thus, Reddit offers a valuable starting place to monitor discussions that may help uncover consumer strategies used to circumvent recent federal and local FTP policies. This study documents how JUUL users learned their preferred pods would be removed from the market, turned to Reddit for information on alternatives, switched to a variety of alternative products, and even gave rise to a new market segment of disposable flavored products. The emergence of DIY communities for mixing flavors [32], legal debates over what is and is not a tobacco product and thus subject to tobacco regulations [33,34,35], and the ability of ENDS manufacturers to adapt to new regulations through unanticipated loopholes [36] highlights the need for expedient means of monitoring policy evasion strategies.

### Limitations and Future Directions

Although this study has many strengths, it is not without limitations. First, the selection biases inherent in social media sampling limits the generalizability of these findings. However, triangulating the findings in this study with data from other sources serves to support these findings, which highlight the quick market shift to disposable flavored brands such as Puff Bar [10]. Secondly, our use of machine learning classifiers to identify flavor policy evasion content within the broader corpus of the whole subreddit lacks precision, since classification algorithms are based on probability and thus subject to misclassification. However, we adhere to rigid criteria for evaluating the quality of these classifiers using both human coders and established conventions, as well as a subsequent qualitative examination of a subsample of posts to provide more nuance than machine coding alone can provide. This study should not be interpreted as an exhaustive examination of FTP policy workaround discussion on Reddit, as we focused on the JUUL-specific subreddit without examining more general ENDS subreddits identified by previous research [37]. Finally, the anonymity of Reddit users limits the generalizability of our findings. However, previous research identified a significant overlap in users of the now-removed r/underageJuul subreddit and r/JUUL [21], strongly suggesting that much of the examined discussion was among young and even teenage users.

## 5. Conclusions

Flavors are consistently identified as the leading reason for tobacco product trial and use, particularly among young people [26,30]. As a result, restricting flavors is an important objective for helping to reduce tobacco use. However, piecemeal approaches to flavor policy are unlikely to be effective [38], as tech-savvy users can turn to an expansive information economy on social media platforms such as Reddit to identify gaps and loopholes in existing FTP policies.

## Figures and Tables

**Table 1:** Most frequently used terms (n-grams) related to flavored electronic cigarette policy restrictions workarounds.

Unigram	*n*	Bigram	*n*	Trigram	*n*
juul	6964	juul pods	1044	juul compatible pods	211
pods	6577	black market	644	black market thc	184
get	3969	mint pods	495	mint juul pods	68
like	3239	mango pods	491	black market carts	66
mint	3224	compatible pods	342	get mango pods	58
mango	2899	juul compatible	341	get mint pods	55
pod	1940	juul pod	222	market thc carts	53
flavors	1770	flavored pods	217	mango juul pods	52
flavor	1762	like juul	215	black market pods	42
menthol	1552	virginia tobacco	214	menthol juul pods	40
one	1441	jewel mint	208	nic salt juice	38
tobacco	1365	juul mint	199	market thc cartridges	38
still	1248	get mango	191	local gas station	35
good	1236	gas station	190	jewel mint diamond	35
would	1235	throat hit	186	3rd party pods	34
pack	1216	market thc	184	juul mango pods	33
juice	1171	juul mango	176	salt nic juice	32
know	1116	mint menthol	172	pods juul compatible	32
people	1114	get mint	166	get flavored pods	31
also	1111	salt nic	163	juul brand pods	31
vape	1098	feel like	162	mr fog pods	31
got	1084	menthol pods	156	flavored juul pods	30
really	1066	taste like	153	local vape shop	28
even	1065	tobacco menthol	144	third party pods	28
nicotine	1033	still get	143	pod juice jewel	28

## Data Availability

The data presented in this study are available on request from the third author. The data are a proprietary of NORC are not publicly available.

## Supplementary Materials

The following supporting information can be downloaded at: https://www.mdpi.com/article/10.3390/ijerph19137668/s1, Table S1. Validated search parameters used to identify posts mentioning flavors and flavor workarounds.
