# Supplementary material for: Discussions of Flavored ENDS Sales Restrictions: Themes Related to Circumventing Policies on Reddit
Source: Int J Environ Res Public Health. 2022 Jun 23;19(13):7668. doi: 10.3390/ijerph19137668 (PMC9266029; doi:10.3390/ijerph19137668)
Supplement: Supplementary file 1 [file ijerph-19-07668-s001.zip › ijerph-1728345-supplementary.pdf]

**Supplementary Table**

**Table S1. Validated search parameters used to identify posts mentioning flavors and flavor workarounds**

| Concept | Keywords and Boolean logic |
|---|---|
| **Flavor**<br><br>Definition: Terms used to determine if a post mentions flavors | Chill, Ice, Icy, Frozen, Cool, Fresh, Mint, Menthol, Arctic, Grape, Grapefruit, Guava, Cucumber, Lime, Lemon, Green, Wine, Razz, Raz, Pink, Berry, Berries, Raspberry, Cola, Soda, Blue, Lychee, Melon, Pineapple, Pina, Colada, Lush, Peach, Banana, Mango, Cream, Blueberry, Red, Pink, Honey, Honeycomb, Honeydew, Pomberry, Peach, Pear, Apple, Vanilla, Cherry, Green, Strawberry, Bourbon, Stout, Whiskey, Rum, Port, Tropic, Tropical, Fusion, Blues, Casino, Deluxe, Jazz, Smooth, Smoothberry, Bird Brains, Boss, Reserve, Unicorn, Cream, Milk, Sugar, Dessert, Cake, Drizzle, Gold, Fruit (y), Watermelon, Rush, OMG, Coffee, Latte, First, Flight, Pomegranate, Medley, Cocktail, Cookie, Wafer, POG, Caramel, Custard, Parfait, Cheesecake, Kiwi, Smoothie, Zest(y), Nectar, Mung Bean, Aromatic, Fragrant, Twist, Blossom, Jasmine, Tea, Crush, Blazing, Vibe, Blast, Passion, Puf, NeWhere, Ne Where, Leap Go, Leapgo, Jak, Bud Vape, Budvape, Bidi Stick, Bidistick, Bidi Vapor, witch Mods, Switchmods, Barz, Cali Bar, Calibar, POP, Hyppe Bar, Hyppebar, MOJO, Puff Bar, Puffbar, Pufbar, Puff Labs, Stix, TWST, SWFT, SWFTbar, SWFTpro, MOTI, MOTIvape, MOTI vape, Supergood, Supergood Bar, Ezzy, Ezzy AF, Ezzyaf, Oval, EzzyOval, Helix, HelixBar, Fog, FogX, FogXVapor, Halo, Vice, HaloVice, Xtra, AirBar, Air Bar, Airbarlux, ORO, UNO bar, Unobar, Bolt, Mana, Manastick, Relx, Thinkr, Drip Stix, Beard Vape, Ryse, Lost Vape, lostvape , Flavor (s/ed/ing/ings/ant/ants), Flavour(s/ed/ing/ings/ant/ants), Taste (s/ed)) |
| **Flavor workarounds**<br><br>Definition: Terms used to determine if a post uses flavor workaround language | flavor(s) AND alternative(s); flavor(s) AND recommend*; "black market"; ship(ping) OR deliver(ing) OR get(ting) OR order(ing) AND flav* AND pods; ship(ping) OR deliver(ing) OR get(ting) OR order(ing) AND (mint OR mango OR cucumber OR abroad); ship(ping) OR deliver(ing) OR get(ting)/order(ing) AND (Canada OR Canadian); juul AND switch(ing); switch(ing) AND cigarette(s); switch(ing) AND iqos; "to the US(U.S.)"; juul AND compatible; juul AND menthol; petition; stig AND flav*; puff AND flav*; posh AND flav*; fog AND flav*; "sea pods" AND flav*; bidi(s) AND flav*; mint OR mango OR cucumber AND "for sale" |

**Note:** For flavors, a list of known flavors was compiled from a variety of places which are represented solely by keywords. For the flavor workarounds, a combination of keywords and Boolean logic was used.
